# Ensemble-based high-performance deep learning models for medical image retrieval in breast cancer detection

**DOI:** 10.1038/s41598-026-38218-y

**Published:** 2026-03-11

**Authors:** Aya E. Fawzy, Mohammed E. Almandouh, Mostafa Herajy, Mohamed Eisa

**Affiliations:** 1 Information Technology Management Department, Faculty of Management Technology and Information Systems, Port Said University, Port Said, Egypt; 2 Mathematics and Computer Science Department, Faculty of Science, Port Said University, Port Said, Egypt; 3 Uruk University, Baghdad, Iraq; 4 Badr University in Cairo (BUC), Cairo11829, Badr, Egypt

**Keywords:** CBMIR, CNN, RNN, XAI, Hybrid model, Cancer, Computational biology and bioinformatics, Health care, Mathematics and computing, Medical research

## Abstract

As digital imaging in healthcare grows quickly, dealing with vast medical image data is getting trickier. Content-Based Medical Image Retrieval (CBMIR) systems help with this, but they struggle because of the gap between simple image details and what these images mean in a clinical setting. This paper presents a new approach using deep learning for CBMIR that combines Convolutional Neural Networks (CNNs), Recurrent Neural Networks (RNNs), and Explainable AI (XAI). Using the Breast Ultrasound Image (BUSI) dataset for training, this hybrid model classifies images and finds the relevant results based on predictions. It reaches a classification accuracy of 99.24% and performs well in retrieval tasks.

## Introduction

Breast cancer is one of the most common cancers among women around the world. It starts in breast tissue and can spread to other body parts. It’s primarily found in the mammary glands and is the second most diagnosed cancer after lung cancer^1^. In 2020, there were about 1.8 million reported cancer cases, with breast cancer making up nearly 30% of that total^2^. Doctors can often spot tumors from breast cancer using medical imaging like X-rays^3^. Tumors can be classified as benign or malignant based on cell characteristics. Catching it early is important for improving survival rates and lowering the chances of death^4^. There are several ways to diagnose and monitor breast cancer, and one of the most popular methods is ultrasound. It is safe, easy to access, and provides real-time images. Unlike other imaging techniques that use radiation, ultrasound uses high-frequency sound waves to create clear pictures of the inside of the body^5^. Since its early application in breast cancer diagnostics^6^, ultrasound technology has seen significant advancements in transducer design, signal processing, and computational systems, significantly improving image resolution and diagnostic precision. It is especially effective in distinguishing fluid-filled cysts from solid masses and, when used alongside mammography^7^, enhances the accuracy of classifying tumors as benign or malignant. Furthermore, ultrasound is commonly used to guide interventional procedures such as biopsies, owing to its real-time imaging and ease of use^8^.

As hospitals started using more diagnostic imaging, they collected many medical images. This rapid increase made managing and finding the right images in big archives tough. Content-Based Image Retrieval (CBIR) helped with this using visual features like color, texture, and shape from the images^9^. These features were turned into high-dimensional vectors, and we check how similar a new image is to those in the database using simple distance calculations, like Euclidean distance. How well CBIR systems work mainly depends on how relevant the features are and how good the similarity measurement is^10^. In the medical field, content-based medical image retrieval (CBMIR) systems have become essential tools for helping doctors with diagnosis, treatment planning, and data management. They provide quick access to similar medical cases, which aids in making better clinical decisions.

Many different methods for automating medical image analysis have been focused at to make these retrieval systems work better and more accurately^11–15^. CBMIR helped with diagnosis and managed large sets of imaging data more effectively^16^. Recent studies have focused into using deep learning for detecting and classifying breast cancer through ultrasound and mammogram images. For instance, in^17^, one study used the Attention U-Net model on the BUSI dataset, with 780 ultrasound images labeled benign, malignant, or normal. This model combines an encoder-decoder structure with attention gates to better localize features and improve segmentation accuracy. It reached an accuracy of 0.98, a precision of 0.97, a recall of 0.90, and a Dice coefficient of 0.92. In another study, Muduli and his team^18^ put forward a deep Convolutional Neural Network (CNN) with five layers to classify breast cancer using mammograms and ultrasounds from various datasets like MIAS, DDSM, INbreast, BUS-1, and BUS-2. The model showed excellent results, scoring 96.55% on MIAS, 90.68% on DDSM, 91.28% on INbreast, 100% on BUS-1, and 89.73% on BUS-2. They also used data augmentation to help improve the model’s performance and reduce overfitting. Jabeen and his team^19^ developed a new method combining deep learning with improved feature selection to classify breast cancer using ultrasound images. They used a few techniques like data augmentation, tweaking a pre-trained DarkNet-53 model, transfer learning, and feature extraction. They applied two advanced algorithms to pick the essential features: Reformed Differential Evaluation (RDE) and Reformed Gray Wolf (RGW). After selecting the features, they combined and classified them using various machine learning methods. When tested on an updated BUSI dataset, their model reached a high accuracy of 99.1%, doing better than previous methods. Notwithstanding significant progress, the current research remains plagued by the problems of having small and single-center datasets, inconsistencies between the datasets, and the lack of cross-dataset validations, all of which cumulatively make it difficult to generalize the model. The underutilization of AI explainability and the absence of clinical validations to any significant degree, along with the resultant high computational complexities, are the factors that currently impede the use of AI in the clinical domain. Future studies need to focus on developing models that are more generalizeable and parsimonious from the computational standpoint.

Key Contribution of this paper:

1. In this paper, we presented a new deep learning setup for classifying and retrieving images related to breast cancer. It combines CNN, RNNs and techniques for XAI to boost accuracy in diagnosis and make the model easier to understand. This approach helped with learning features and ensured that the outcomes could be interpreted, which was necessary for making decisions in a clinical setting.
2. The proposed model made good use of the strengths of each part in the hybrid model, we used CNN for spatial feature extraction, RNN for modeling sequential dependencies and XAI to visually explain the model’s predictions and gaining trust in clinical settings, helping doctors understand why the model made certain decisions.
3. We used data augmentation methods to help the model work better in different situations and avoid overfitting due to insufficient medical images. That is, we rotated, flipped, scaled, and adjusted the contrast of the images. By adding more variety to the training set, the model got better at handling differences it might see in actual clinical data.
4. We used Euclidean distance for CBIR module to measure similarity. After classifying the images, we compared deep features using this distance to find visually and semantically similar images in the dataset. It helps radiologists by providing them with related cases for reference during diagnosis.
5. This paper is set up like this: In Sect. 2, we provided a review of related work. Section 3 goes over the methods we used. Section 4 shares the results and how we evaluated them, and Sect. 5 wraps things up with conclusions and ideas for future work.

## Related work

There are more medical imaging data out there these days, and systems like Picture Archiving and Communication System (PACS)^20^ are being used more than ever, which means we need innovative tools to help retrieve images and diagnose. In particular, when it came to spotting breast cancer, deep learning models were stepping up as strong options for automating the diagnosis and making sense of ultrasound and mammogram images. That said, it could still be tough to pick up on small details and make sure AI predictions were straightforward to understand.

Pretrained CNNs were popular for boosting performance and making training easier. Several previous studies had paired these models with explainable AI techniques like Gradient-weighted Class Activation Mapping (Grad-CAM) to make things more straightforward. Masud and his team (2022) used ResNet50 and VGG16 models to classify breast ultrasound images. They got an accuracy of 92.4% and an area under the curve (AUC) of 0.97. They used Grad-CAM visualizations to show which areas were necessary for their classifications, making it easier to understand the results^21^. In another study, Dong and his team (2021) applied DenseNet-121 to identify lesions in ultrasound images. Their model reached an accuracy of 88.4% and had good sensitivity and specificity. Grad-CAM also helped reveal the important clinical areas during their analysis^22^. Finally, Suh and his team (2020) looked at DenseNet-169 and EfficientNet-B5 to find malignant cases, reporting accuracies of 88.1% and 87.9%, respectively. Their Grad-CAM visuals focused on tumors and nearby tissues, showing how crucial context is in these cases^23^.

Researchers in previous works improved model designs by using attention mechanisms and multi-branch structures to find essential features more effectively. Lou and his team (2022) devised a new way to analyze mammograms using a multi-level global-guided branch-attention network (MGBN). It got AUC scores of 0.8375 on the DDSM dataset and 0.9311 on INbreast. They used Grad-CAM to check how well the model focused on areas with masses^24^. Building on that, Wang and his team (2023) introduced MIB-Net, which was a multitask model that did both tumor classification and segmentation. They also used Grad-CAM to ensure their predictions were zeroing in on the essential parts of the images^25^. Some studies compared different CNNs or created team strategies to improve diagnostic accuracy. Ahmed and his team (2021) looked at different transfer learning models like VGG16, InceptionV3, ResNet18, and ResNet50 using mammogram data. They split the dataset similarly for fair comparisons and got as high as 95% accuracy^26^. Raza and his team (2022) developed a custom model called Deep Breast Cancer Net, which has 24 layers and uses convolutional layers, inception modules, and special activation functions. It hit 99.35% accuracy on the primary dataset and 99.63% on another dataset, beating the other models out there^27^.

Previous researches looked at how CNNs stack up against regular machine learning algorithms. Cruz-Roa and his team (2014) used a CNN to spot invasive ductal carcinoma (IDC) in whole slide images of tissue samples. They trained their model on 113 slides and tested it on 49, getting an F1 score of 71.8% and a balanced accuracy of 84.2%. This shows that the model performed well in detecting localized cancer^28^. Then, Yap and his team (2018) applied the Faster R-CNN model to find breast masses. It showed excellent results with a recall of 0.9236, a precision of 0.9408, and an F1 score of 0.9321. They found that using RGB images helped improve the model’s performance, but its high computational needs could make it tough to use widely^29^. Zhao and his team (2019) took a different approach and used YOLOv3 to locate and size lesions from the Stanford Mammography dataset. Their model hit an F1 score of 0.88, proving that real-time detection methods work well for clinical images^30^. In (2021), Sun and his team trained CNNs on ultrasound data, scoring an AUC of 0.72, which showed decent performance. They pointed out that they did not use cross-validation, which suggests there’s room for better evaluation methods^31^. Then in (2022), Sun and his team compared CNNs with Random Forest classifiers using a larger set of 2,395 ultrasound images. The CNNs outshone the Random Forests, with the best AUC reaching 0.912 when looking at intertumoral and peritumoral areas^32^.

Some studies suggested that combining different deep learning methods could help make breast cancer detection more accurate and easier to understand. Salman and his team (2021) developed a hybrid model that mixed granular computing, shortcut connections, learnable activation functions, and attention mechanisms. Granular computing helped break down complex data into smaller pieces, making it easier to learn detailed features. This model reached an accuracy of 93% with ultrasound images and 95% with histopathology images. It’s effective, but its complexity might make it hard to scale in real clinical settings^33^. On the other hand, Sakthivel and his team (2022) introduced a hybrid CNN-RNN model to analyze patterns over time in mammogram images. They tested it on the DDSM dataset, which has 9,000 images, and showed a sensitivity of 90%, proving it’s good at analyzing images in sequence. Still, more testing with real clinical data is needed^34^.

Numerous investigators have investigated ensemble models and transfer learning to improve how well models work and adapt. Bejnordi and his team (2017) developed seven deep learning algorithms to spot lymph node metastases. The best model hit an impressive AUC of 0.99, beating human pathologists with an AUC of 0.810. This showed how AI could help in critical diagnostic situations^35^. Then, Hekal and his team (2021) created an ensemble model that mixes four pretrained CNNs with an SVM classifier. This setup reached 94% accuracy for detecting malignant cases and 95% for benign ones on the CBIS-DDSM dataset. It’s a strong approach, but it did not make things more complicated from a tech perspective^36^. Shah and his team (2023) worked on another ensemble using EfficientNet, AlexNet, ResNet, and DenseNet, adding features like variable dropout and weighted skip connections. Their model got a precision of 94.6%, sensitivity of 92.4%, specificity of 96.1%, and an AUC of 98.0%. This showed great promise for catching issues early, though the complexity of the design could make it tough to roll out^37^.

Karthik and his team created a new Computerized Decision Support System (CDSS) to address the deficiencies associated with EHR-based systems by introducing a type of system that utilized Content Based Medical Image Retrieval (CBMIR). The basis for their framework was centered around utilizing artificial intelligence (AI) medical image classification from multiple sources of imaging data in order to enhance identification of and support for diagnosis and treatment of diseases. By integrating classification based filtering and tree based similarity matching to facilitate more rapid processing and retrieval of all medical images, they achieved Improved Mean Average Precision scores of 0.66 to 0.85 through Top-5 Retrieval Systems with an overall time complexity of O(logn)^38^. Karthik et al. developed an adversarial training framework that combines generative adversarial networks (GANs) with convolutional neural network (CNN)-based multi-label classification for chest X-ray analysis. Using the NIH Chest X-ray dataset, their method showed that adversarially trained CNN multi-label classifiers worked better, with an AUC gain of about 6% compared to regular standalone CNN models. they then used a CNN-RNN (CNN-LSTM) framework to evaluate the acquired representations for description generation. they found that adversarially trained features led to more accurate and relevant image descriptions. The study employed a pre-trained VGG-16 model as the discriminator, noting training instability and underscoring the need for further tuning. The results show that adversarial generative training makes classification more accurate, reduces overfitting on small datasets, and can be improved even more with attention mechanisms, self-attention GANs, and transfer learning^39^. Although we have made significant progress, several issues remain. Many models have been tested on small datasets from a single center, and while some studies have utilized Grad-CAM, the use of explainable AI should be more widespread. Complex models may pose challenges in settings with limited resources. Additionally, few studies have evaluated these models within real-world hospital systems or clinical trials.

In Table 1, we compared earlier methods and demonstrated how deep learning can enhance the accuracy and clarity of breast cancer diagnostics. Tools like Grad-CAM and attention mechanisms have helped explain decision-making processes of the models; however, challenges still exist, such as variations in datasets and the need for validating results across different studies. For future research, it would be great to develop models that work well with various patient groups and imaging types, and also found ways to make them easier to understand in a clinical setting.

**Table 1:** Comprehensive comparison of recent studies using deep learning for breast cancer detection with medical images. Each one showed the data they used, the deep learning setup, the advantages and disadvantages for their models.

Study	Dataset	Method	Advantages	Disadvantages
Masud et al. (2022)	Breast Ultrasound Images (BUSI)	ResNet50, VGG16 + Grad-CAM	High accuracy (92.4%), AUC (0.97), interpretable via Grad-CAM	Validation is limited across different datasets.
Dong et al. (2021)	2D Ultrasound Dataset	DenseNet-121 + Grad-CAM	Balanced accuracy, sensitivity, specificity	Reduced accuracy in specific regions.
Suh et al. (2020)	Not specified	DenseNet-169, EfficientNet-B5 + Grad-CAM	Highlights tumor + surrounding tissue	A small drop in accuracy.
Lou et al. (2022)	DDSM, INbreast	MGBN + attention + Grad-CAM	Strong AUC (0.8375, 0.9311); interpretable	Complex models require a lot of computing power.
Wang et al. (2023)	Multi-modal (Ultrasound + others)	MIB-Net multitask + Grad-CAM	Combines classification + segmentation	Lack of external validation
Ahmed et al. (2021)	Mammographic dataset (not named)	VGG16, InceptionV3, ResNet18/50 (transfer learning)	Up to 95% accuracy; consistent benchmarking	Does not evaluate how easy it is to understand.
Raza et al. (2022)	Two unnamed datasets	DeepBreastCancerNet (custom DL model)	High accuracy (99.35–99.63%); optimized structure	Complex models
Cruz-Roa et al. (2014)	Whole Slide Images (WSI)	CNN for IDC detection	Balanced accuracy (84.2%), localized tumor focus	F1 score moderate, but it hasn’t been checked with outside data.
Yap et al. (2018)	Various mammogram datasets	Faster R-CNN	High recall/precision; RGB improves performance	High computing expenses.
Zhao et al. (2019)	Stanford Mammography	YOLOv3 (real-time detection)	Real-time lesion detection; F1 score: 0.88	Interpretability limitations
Sun et al. (2021)	248 ultrasound images	CNN	Introduces CNN for US; 72.6% accuracy	The AUC score is 0.72, but cross-validation was not done.
Sun et al. (2022)	2,395 ultrasound images	CNN vs. Random Forest	CNN AUC = 0.912; outperforms RF	Unclear CI methods; validation approach unspecified
Salman et al. (2021)	Ultrasound + Histopathology	Hybrid (granular computing, shortcut, attention)	High accuracy (93–95%); flexible architecture	Hard to scale due to design complexities.
Sakthivel et al. (2022)	DDSM (9,000 images)	Hybrid CNN-RNN	Models’ temporal patterns; 90% sensitivity	Lacks real-world clinical validation.
Bejnordi et al. (2017)	Camelyon16	Ensemble of 7 DL models	Top model AUC: 0.994; exceeds pathologists	Interpretability not analyzed
Hekal et al. (2021)	CBIS-DDSM	Ensemble (4 CNNs + SVM)	High binary accuracy: 94–95%	Ensemble complexity; computational cost
Shah et al. (2023)	Custom (early detection)	Ensemble: EfficientNet, AlexNet, ResNet, DenseNet	AUC: 98%, precision: 94.6%, strong generalization	Complex ensemble; harder to deploy
Karthik et al. (2024)	Multi-modal medical imaging datasets (X-ray, MRI, CT)	CDSS worked with CBMIR by using AI-based image classification, classification-based filtering, and tree-based similarity matching.	Improved evidence-based diagnosis by using medical images; had a high retrieval rate (MAP 0.66–0.85 for Top-5) and retrieves quickly with logarithmic time complexity. O(logn)	Primarily focused on retrieval performance; limited discussion on deep feature learning robustness and generalization across diverse clinical scenarios
Karthik et al. (2025)	NIH Chest X-ray dataset	Adversarial training framework combining GANs with CNN-based multi-label classification; CNN-LSTM for image description generation	Improves multi-label classification performance with ~ 6% AUC gain; reduces overfitting on limited datasets; generates more accurate and relevant image descriptions	GAN training instability when using pre-trained VGG-16 as discriminator; requires further optimization; attention mechanisms and self-attention GANs not fully explored

## Methodology

### Dataset

In 2018, A Breast Ultrasound Image (BUSI) dataset^40^ featuring 600 women aged between 25 and 75 was collected. This dataset included 780 ultrasound images, each around 500 × 500 pixels, and saved in PNG format. The images fall into three groups: normal, benign, and malignant. The breakdown of images is shown in Table 2.

The ultrasound images in this paper were in grayscale and were first collected in DICOM format at Baheya Hospital^40^. It took about a year to gather and label the images. The dataset was split into three groups: normal, benign, and malignant. While we started with 1,100 images, we trimmed it down to 830 after preprocessing to remove parts that did not help with classifying masses and might mess with training the model.

**Table 2:** Distribution of 830 breast ultrasound images: most of them, about 62.4% (487 images), are benign, while 26.9% (210 images) are malignant, and the remaining 10.7% (133 images) are normal.

Categories	Number of images
Benign	487
Malignant	210
Normal	133
Total	830

### Dataset preprocessing

We made some tweaks to improve the dataset quality and usability. First, we found and removed duplicate images. We changed the original DICOM files into PNG format using a conversion tool^41^. After all that, we ended up with 830 ultrasound images. We organized these images into three groups: normal, benign, and malignant. To get rid of unnecessary background areas, we cropped all images to different sizes using the Fast Photo Crop tool. Then, we resized all the ultrasound images to 150 by 150 pixels for consistency. We also normalized the pixel values to be between 0 and 1, which helps with training the model. We included annotation details in the image filenames for easy reference.

**Fig. 1:**
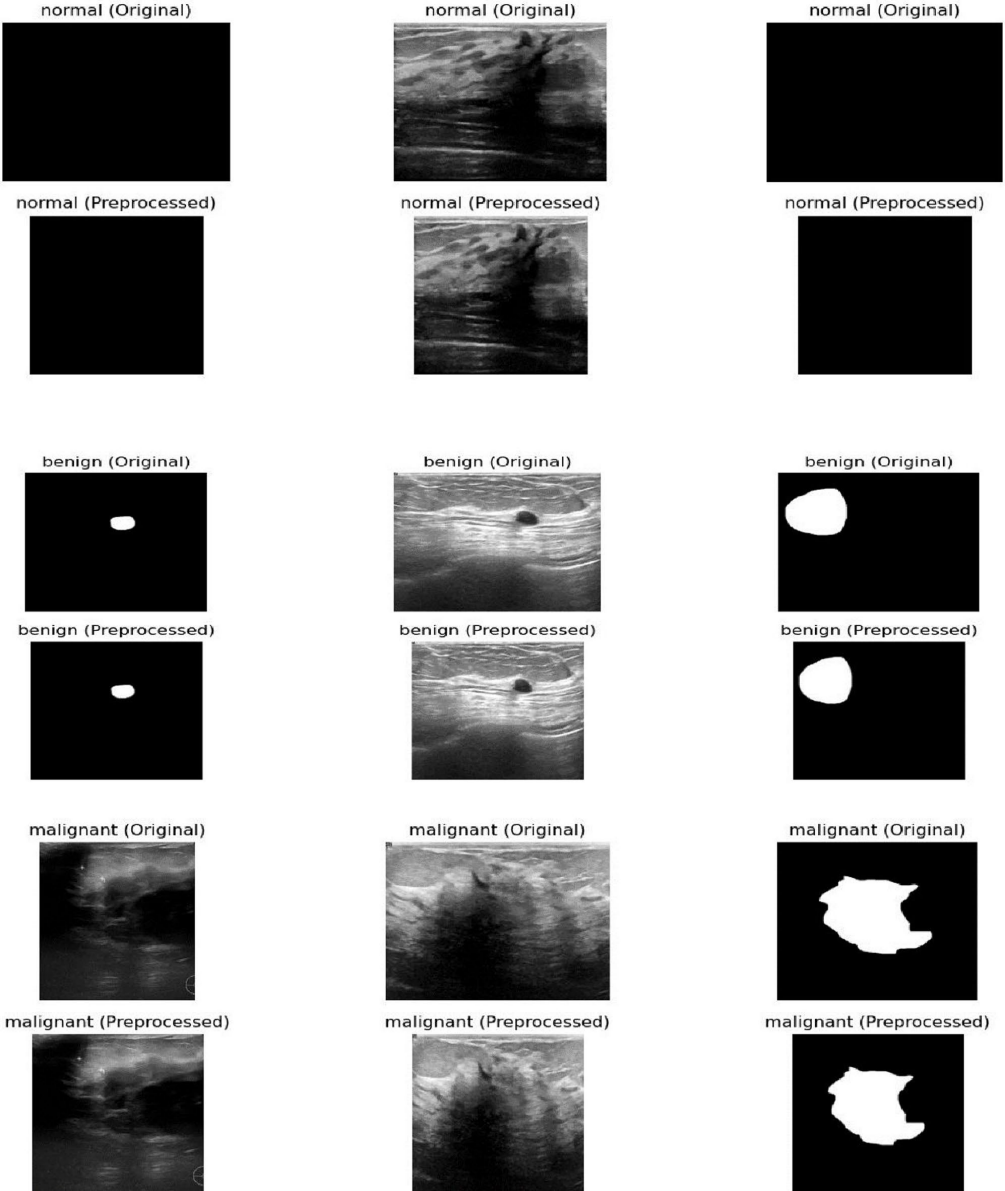
Comparison between the original breast ultrasound images (top row) and the preprocessed breast ultrasound images (bottom row) for normal, benign, and malignant cases. The preprocessing steps are converting the images to grayscale, enhancing the contrast, and resizing them to improve image quality and make them uniform in size for input to the model. Additionally, feature extraction, feature representation, and latent space embeddings are essential steps that follow preprocessing to enable accurate retrieval and classification, but they are not shown in this figure.

Figure 1 shows a comparison between the original breast ultrasound images (top row) and the preprocessed images (bottom row) for normal, benign, and malignant cases. While the figure demonstrates the visual improvements from preprocessing, the original explanation misses several critical steps in the workflow:

- **Preprocessing**: The images were cropped to remove unnecessary background areas using the Fast Photo Crop tool and then resized to 150 × 150 pixels to maintain consistency. Pixel values were normalized between 0 and 1 to facilitate model training.
- **Feature Extraction**: After preprocessing, relevant features from the images were extracted, such as texture, shape, and intensity patterns, which are essential for distinguishing between normal, benign, and malignant cases.
- **Feature Representation**: The extracted features were represented in a structured format suitable for input to the deep learning model, enabling efficient processing and learning.
- **Latent Space Embeddings**: The model transformed the feature representations into a latent space, where images with similar characteristics are positioned closer together. This embedding helps the model capture complex relationships between different image types.
- **Retrieval Procedures**: Finally, these embeddings enable efficient retrieval and classification of new ultrasound images by comparing their latent representations with those of the training set.

To enhance the ultrasound dataset for diagnostic purposes, we manually annotated critical areas in each image using Python with a freehand method. We organized the dataset into three folders based on the diagnosis types: normal, benign, and malignant. Each image had a clear naming system that showed its class and a number. The mask files kept the same name as the original images but had a mask at the end to show they were segmented. In these masks, each pixel was labeled as either 0 for non-cancerous areas, like healthy tissue or background, or 1 for cancer-affected regions. We split the dataset into two parts: 80% of the images were used for training the model, and the other 20% were set aside for testing. This way, we could see how well the model performs on data it hadn’t seen before, giving us a better idea of how well it worked in real situations.

### Modelling of dataset classifiers and feature extraction

Deep Learning Models used for Medical Image Analysis provide a method of extracting certain characteristics, or features, from medical images, creating viable, reliable classifiers of medical images through the development of feature extraction algorithms that convert digital medical images into usable data sets. Through various deep learning techniques, including CNN, RNN, XAI and hybrid models, the model uses knowledge from previous analyses or ‘intents’ to identify emerging patterns and build more advanced machine learning processes. In this section, you will learn about each of the deep learning models used, the feature extraction algorithms used for each, and the classifier models that were created through use of the training dataset.

### Deep learning model architectures (CNN, RNN, XAI, Hybrid)

There is a wide variety of deep learning architectures available to assist with medical image interpretation. Deep neural networks are comprised of many layers (CNN, RNN, and Hybrid) which provide different ways to analyze images. In health care we can use CNN for image recognition and texture features extraction, RNN for time-dependent modelling, Explainable AI (XAI) to improve the ability to understand how a particular result has been reached through use of deep learning and Hybrid systems that draw upon these three models.

#### CNN model architecture

We used the deep learning method CNN to learn and identify important features in different medical images. Medical images usually come as 3D data made up of 2D slices taken from the area we are studying. First, we trained the CNN to classify these images. After that, we used what we had learned to help with finding similar medical images based on their content. We also checked how well this setup works utilizing a dataset that includes various imaging methods and organs^42^. Convolutional Neural Networks, or CNNs, were built with different layers that work together^42^. They have convolutional layers that pick out local features using filters that could be trained, and pooling layers that help to simplify the data by grouping nearby features^43^. This setup helped making the model less sensitive to small changes. How well CNNs perform in classifying data really depends on how these layers were arranged and the training methods used^44^.

#### RNN model architecture

Recurrent Neural Networks (RNN) and CNN were both good at pulling important details from image data. They played similar roles in the overall setup of the model. It is widely recognized that images inherently possess spatial properties. However, when examined at the pixel level, a sequential dependency could also be observed among pixel values. By interpreting the image width as the feature dimension and the height as a temporal sequence, each horizontal line of pixels could be conceptualized as a time step within a sequence. This perspective enabled the modeling of both spatial and temporal dependencies within an image^45^. RNN, come in different types, like globally recurrent, locally recurrent, time-delayed, and simultaneous recurrent networks^46^. They usually had two main setups: fully connected and partially connected. A critical part of RNNs was the hidden state, which changed with each step based on the current input and the previous hidden state^47^. his mechanism enables the network to track relationships over time within sequences.

#### XAI model architecture

Explainable Artificial Intelligence, or XAI, is all about creating systems that not only make accurate predictions but also help folks understand how those predictions were made. This way, users can trust the models behind the scenes. By using XAI in machine learning, we can ensure that models are more reliable, spot causal patterns in data, and help people make better, informed decisions. It also boosts ethical responsibility since it makes everything clearer and more understandable^48,49^.

XAI methods could be split into two main types: models that were easy to understand from the start and those that needed extra steps to explain their decisions. The first type included models like decision trees and K-Nearest Neighbors, which were straightforward and clear^50^. The second type was for more complex models, like deep learning tools such as Convolutional Neural Networks (CNNs) and Recurrent Neural Networks (RNNs). These often-needed additional techniques help explain how they made their choices^51^.

Post-hoc explanations could be split into two types: global and local. Global interpretability looks at the big picture of how the model works, covering things like how it was built, how it was trained, and what kind of data was used. Local interpretability zooms in on specific predictions, helping to show which input features impacted those particular results^52^.

Post-hoc techniques could be divided based on whether they depend on the model’s structure. There were model-specific techniques, like Grad-CAM, which worked with certain types of models and couldn’t be used with others. On the flip side, there were model-agnostic methods, such as LIME and SHAP, which are flexible and can be used with many different models.

#### Hybrid-based model architecture

To improve how static images were understood, this paper used a new hybrid model that combines RNNs with CNNs. RNNs usually worked best with data in a sequence, so we tweaked them to work with static images by breaking each image down into non-overlapping patches. We flattened these patches and lined them up in a sequence, which let the RNN look at the relationships between different patches, like it did with time-based data. This way of processing helped the model pick up on essential details and patterns in the image, making it better at tasks like classifying and retrieving images.

To help make sense of deep learning predictions, we used Grad-CAM, which stands for Gradient-weighted Class Activation Mapping. This method created heatmaps showing which parts of an image were most important in the model’s decision. This visual aided increase transparency and built trust, making sure the model was looking at the right areas for diagnosis and classification. In the image retrieval part, we got feature vectors from the layer right before the softmax activation in the hybrid model. These vectors hold essential details about the images and help represent them in a specific way. When we need to find similar images, we calculate the Euclidean distance between the feature vector of the image we’re querying and the ones in the database to see which ones are the closest matches.

Data augmentation is very helpful when we have a small original dataset and want to avoid overfitting. Some common techniques were rotating images, flipping them horizontally and vertically, scaling, and improving contrast. After using these techniques, the training set grows from 548 images to 3,840 images, which makes the model stronger and more reliable. We then divide this larger dataset into 80% for training, 10% for validation, and 10% for testing. In this new hybrid deep learning setup, we performed image retrieval using deep features from a trained classification model. We pulled features from the second-to-last layer of the hybrid model, right before the last softmax classification layer.

This layer produced a detailed feature vector that holds essential information about the input image. Once the image went through the previous convolutional layers, RNN, and XAI sequence processing, the network picks up on complex patterns and relationships that were key for breast cancer classification. These feature vectors acted like unique IDs for images, putting each one in a high-dimensional space. This layer was designed to separate different classes, so it grouped similar images close to each other, which was helpful for finding images later. To retrieve images, we got the feature vector for a query image using a hybrid model we trained. Then, we calculated the Euclidean distance between this query vector and all the feature vectors in the database. Finally, we ranked the images by distance, pulling out the ones that are most similar to the query based on high-level features. This method ensured that retrieval was not just based on basic visual traits like pixel brightness or texture. Instead, it focused on learned features like tumor shape, how well the boundaries were defined, and tissue structure, which really matter for clinical decision-making.

**Fig. 2:**
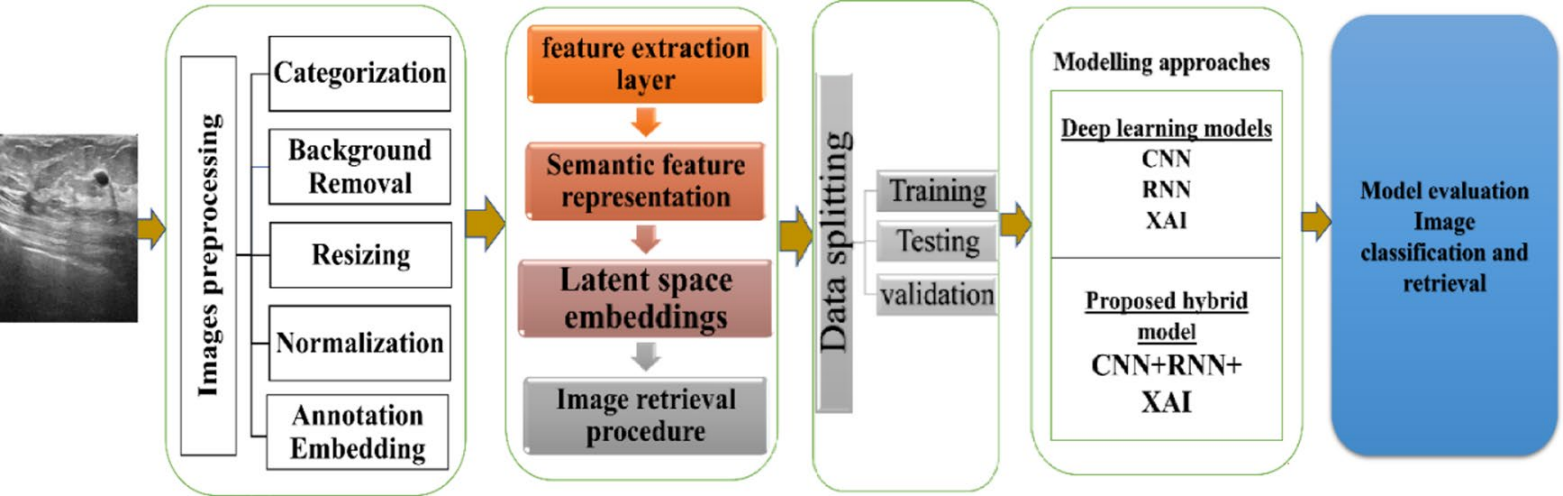
Proposed hybrid deep learning framework for breast cancer image classification and retrieval which presented how the model work. First, the images are preprocessed that involved sorting, removing backgrounds, resizing, making the images uniform, and adding notes to make the input data standard and the important parts clearer. Then, the preprocessed images go through a step where features are extracted and put into a hidden area for better representation. Finally, they were used to sort the images and find similar images.

Using the second-to-last layer for getting features, the model struck a good balance between the depth of the representation and the relevance of the results. This helped improve performance in classifying images and finding them based on content. To make our hybrid model easier to understand, we used Gradient-weighted Class Activation Mapping, or Grad-CAM. This technique was popular for explaining how convolutional neural networks work. Grad-CAM created heatmaps that show which parts of the input image were most important for the model’s decision. Grad-CAM worked by comparing the gradients of the score for a predicted class to the feature maps from the last convolutional layer. It averaged these gradients to get weights for each channel, which were then combined with the feature maps. A ReLU activation was used to keep only the features that matter for the target class. It gives us a rough localization map that’s resized and placed on top of the original image, showing the parts that played a significant role in the prediction. In this paper, we used Grad-CAM on the CNN part of the model to check if it focused on critical areas like tumors or unusual tissue patterns in breast ultrasound images. It helped clinicians understand how the model worked, which built their trust in the AI for detecting breast cancer. Figure 2 shows the setup of the hybrid model.

### Deep learning models hyperparameters

#### CNN hyperparameters

We reviewed the hyperparameters used when developing and training a CNN for classifying breast cancer images. We organized these hyperparameters into three main categories: preprocessing, model design, and training setup, which are presented in Table 3.

During the preprocessing stage, we resized each image to a standard size of 150 × 150 pixels so everything matched up in the dataset. We also changed the images to grayscale using the.convert(‘L’) function, which made the data simpler by cutting down the color channels to just one. Then, we normalized the pixel values to be between 0 and 1 by dividing all the numbers by 255.0. It helped speed up training and kept things stable.

The CNN model had a simple structure made up of four convolutional layers with filter sizes of 32, 64, 128, and 256, all using ReLU activation to add some non-linearity. After each convolution, MaxPooling2D with a (2, 2) kernel is applied to reduce the size of the feature maps and help prevent overfitting. After the convolutional part, there are two fully connected layers: the first has 512 units, and the second has 256 units, and they both use ReLU activation. To further reduce overfitting, there is a dropout layer set at 0.3 that randomly turns off neurons during training. Finally, the output layer has 3 neurons with a Softmax activation function to predict probabilities across three different classes.

We used the Adam optimizer to train the model, which is known for being efficient and easy to set up. We kept the learning rate at the default of 0.001. For measuring loss, we went with categorical cross entropy since it works well for tasks with multiple classes.

We measured the model’s performance mainly by looking at accuracy. Training was done with a batch size of 32 samples and a maximum of 15 epochs. We also set aside 10% of the training data for validation. Plus, we implemented early stopping with a patience of 2 epochs to keep an eye on validation accuracy, so training would stop if it did not improve after a couple of tries.

**Table 3:** Hyperparameter configuration and model architecture for CNN model used in breast cancer ultrasound image classification into benign, malignant, and normal categories.

	Category	Hyperparameter	Value	Description
CNN model	Preprocessing	Image Size	(150, 150)	Input dimensions for CNN
		Color Mode	Grayscale	Converted using.convert(‘L’)
		Normalization	/255.0	Normalize pixel values to [0, 1]
	Model architecture	Conv Layer 1 Filters	32	First convolution layer with 3 × 3 kernel
		Conv Layer 2 Filters	64	Second convolution layer
		Conv Layer 3 Filters	128	Third convolution layer
		Conv Layer 4 Filters	256	Fourth convolution layer
		Activation Function	ReLU	Used in all hidden layers
		Pooling Type	MaxPooling2D	Downsampling operation
		Pooling Size	(2, 2)	Pooling kernel size
		Fully Connected Layer 1	512 units	Dense layer with ReLU activation
		Dropout Rate	0.3	Applied after dense layers
		Fully Connected Layer 2	256 units	Another dense layer
		Output Layer	3 units, Softmax	For 3-class classification
	Training configuration	Optimizer	Adam	Adaptive optimizer
		Learning Rate	Default (0.001)	Used by Adam unless manually changed
		Loss Function	Categorical Crossentropy	Suitable for multi-class classification
		Metrics	Accuracy	Evaluation metric
		Batch Size	32	Number of samples per training batch
		Epochs	10	Maximum training iterations
		Validation Split	0.1	10% of training data used for validation
		Early Stopping	Patience = 2	Monitors validation accuracy for improvement

#### RNN hyperparameters

This paper used RNN to sort breast tumor images into three groups: benign, malignant, and normal. We trained the model on grayscale images that we resized and adjusted before putting them into the network. Table 4 details the hyperparameter setup we used during the development and training. We resized the input images to 150 × 150 pixels and made them grayscale, turning them into single-channel images. This helped reduce the complexity and processing time while still maintaining enough detail for classifying tumors. We also normalized the pixel values to fit between 0 and 1, which helped the training process run smoother by making the data more consistent. After the convolutional layers, the features were reshaped and sent to an LSTM layer. The LSTM had an input size of 64 × 37 × 37, which came from the size of the pooled feature maps. It had a hidden state size of 128 and just one recurrent layer. We turned on the batch_first option to make sure the input format matches what PyTorch used. The LSTM’s last output went through a dense layer that changed the 128 dimensions into a 3-dimensional vector. This vector showed the probabilities for each class for the images. By using this thick layer, the model could turn the LSTM’s time-based patterns into scores that help with classification. We trained the model using the Adam algorithm with a learning rate of 0.001. For the loss function, we went with categorical cross-entropy since it worked well for multi-class classification. The training lasted for 10 epochs with a batch size of 32, making the process efficient for computing and updating gradients. We split the data into training and testing sets in a 80:20 ratio. To keep things consistent, we set a random seed at 42 and used stratified sampling to keep the class distribution the same in both sets.

**Table 4:** Hyperparameter configuration and model architecture for RNN model used in breast cancer ultrasound image classification into benign, malignant, and normal categories.

	Category	Hyperparameter	Value
RNN model	Image preprocessing	Image size	150 × 150
		Color mode	Grayscale
		Normalization	Pixel values scaled to [0, 1]
	RNN (LSTM)	Input size	64 × 37 × 37 = 87,361
		Hidden size	128
		Number of layers	1
		Sequence format	batch_first = True
	Fully connected layer	Input size	128
		Output size (classes)	3
	Training	Optimizer	Adam
		Learning rate	0.001
		Loss function	CrossEntropyLoss
		Batch size	32
		Number of epochs	10
	Dataset Split	Train/Test split	80%/20%
		Random seed	42
		Stratified sampling	Yes

#### XAI hyperparameters

In This paper, we used a transfer learning method based on the MobileNetV2 architecture, which was already trained on the ImageNet dataset for our image classification model. This part gives an overview of the hyperparameters and design decisions we made for preprocessing, building the model, and training it, as shown in Table 5.

All the input images were resized to 150 by 150 pixels to meet the MobileNetV2 model’s requirements. Before putting the images into the network, we normalized the pixel values to a range of 0 to 1. It helped make the training process smoother and faster. To help the model learn better and avoid overfitting, we used some data augmentation techniques in real time. This included random rotations up to 30 degrees, moving the images left or right by up to 20% of their width, shifting them up or down by 20% of their height, applying shear transformations of 0.2, zooming in and out a little, and flipping the images horizontally. Moreover, we set aside 20% of the dataset for validation while loading the data.

The model was built on MobileNetV2, which was a lightweight convolutional neural network designed for mobile and small devices. For this setup, we left out the top layers of the original model and kept the pre-trained base unchanged to maintain the features it had learned. We then added a global average pooling layer to shrink the dimensions while keeping the key features. After that, we set up a fully connected layer with 256 units and used a ReLU activation function. To help avoid overfitting, we included two dropout layers, each with a dropout rate of 0.5, one right after the pooling layer and another after the dense layer, which randomly turns off some neurons during training. Finally, the last layer used a softmax activation function to give probabilities across three categories: benign, malignant, and normal.

The model was assembled using the Adam algorithm, which adjusts the learning rate as needed. We used sparse categorical cross entropy for the loss function because it worked well for multi-class problems and integer labels. To see how well the model performed, we tracked the classification accuracy. Training was done in mini-batches of 32 samples over 10 epochs. To keep overfitting in check and help with learning, we added two callbacks. One was an early stopping callback that monitored validation loss and stopped training if there was not any improvement for five epochs. The other callback reduced the learning rate by 0.2 if the validation loss stayed the same for three epochs, with a minimum learning rate set at 1 × 10⁻⁶.

**Table 5:** Hyperparameter configuration and model architecture for XAI model used in breast cancer ultrasound image classification into benign, malignant, and normal categories.

	Category	Hyperparameter	Value
XAI model	Image preprocessing	Target image size	150 × 150
		Rescaling	1./255 (normalization)
		Augmentation: rotation range	30 degrees
		Augmentation: width shift	0.2
		Augmentation: height shift	0.2
		Augmentation: shear range	0.2
		Augmentation: zoom range	0.2
		Augmentation: horizontal flip	True
		Validation split	0.2 (20%)
	Model architecture	Base model	MobileNetV2 (pre-trained on ImageNet)
		Include top	False
		Trainable base	False (frozen)
		Global Average Pooling	Yes
		Dense layer 1	256 units, ReLU activation
		Dropout (after pooling)	0.5
		Dropout (after Dense)	0.5
		Output layer	3 units, Softmax activation
	Training	Optimizer	Adam
		Loss function	Sparse Categorical Crossentropy
		Metrics	Accuracy
		Batch size	32
		Epochs	10
	Callbacks	Early stopping patience	5 epochs
		Reduce LR patience	3 epochs
		Reduce LR factor	0.2
		Minimum learning rate	1e-6

#### Hybrid deep learning mode hyperparameters

**Fig. 3:**
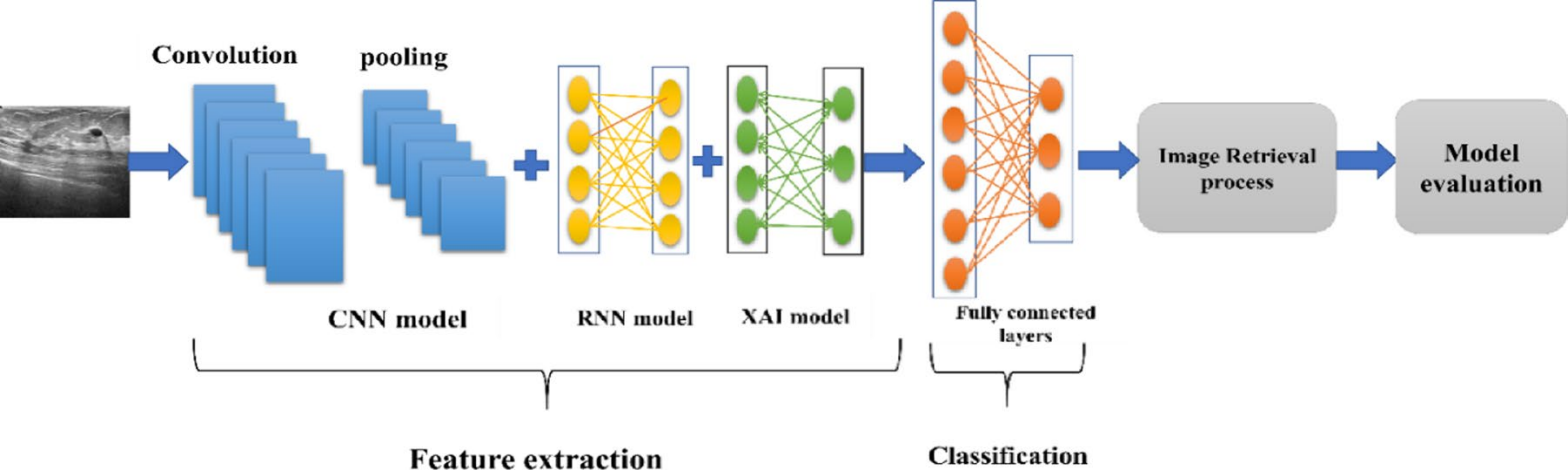
Architecture of proposed hybrid deep learning model for breast ultrasound image classification and retrieval which used CNNs, RNNs, and XAI. This model is made to classify breast ultrasound images and to help find similar images.

This proposed hybrid model classified images using transfer learning with the VGG16 neural network. It focused on sorting medical images into different diagnostic categories. Below, we have laid out the choices for hyperparameters that we used during preprocessing, building the model, and training. Table 6; Fig. 3 present a clear look at these details, while Algorithm 1 shows how the hybrid deep learning model works.

**Algorithm 1:**
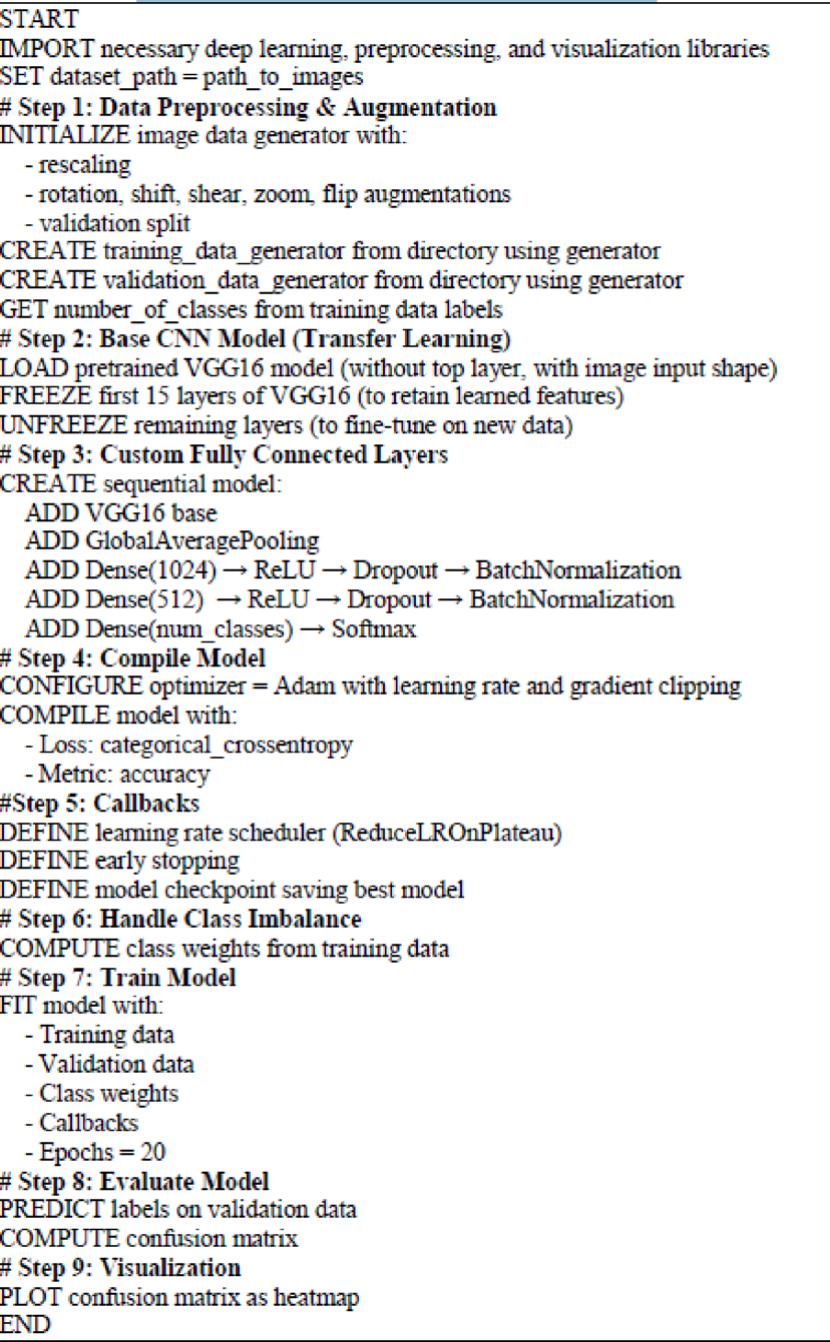
Proposed hybrid deep learning model algorithm.

All images were resized to 150 × 150 pixels. The pixel values were adjusted to be between 0 and 1 by dividing by 255 to keep everything stable during training.

To boost the dataset and help the model perform better, we used some data augmentation tricks. These included rotating the images randomly by up to 20 degrees, shifting them horizontally and vertically by up to 20%, shearing and zooming within a 0.2 range, and flipping them horizontally. This augmentation happened on the fly while training with a data generator. Plus, 10% of the data was set aside for validation.

We loaded the images in batches of 64 and classified them with one-hot encoding for the labels. This setup allowed us to handle multiple classes and was better for memory use while training the model.

The model used the VGG16 setup as a base and had weights from the ImageNet dataset. It left out the top layers of the original network and only made the last few convolutional layers trainable so it could fine-tune those parts. The other layers were fixed to keep the general features learned from the original dataset.

On top of VGG16, we added more fully connected layers for classification. A global average pooling layer was used to shrink the spatial size. Then, we included two dense layers with 1024 and 512 units, both using ReLU for activation. To prevent overfitting, L2 regularization was applied to those layers, with a penalty factor of 0.0001. We also used dropout rates of 0.4 and 0.3 after each dense layer, along with batch normalization to help with learning stability.

Finally, the last classification layer had a softmax activation function, which gave output probabilities based on the number of target categories.We set up the model using the Adam optimizer and chose a learning rate of 0.0005. To avoid issues with exploding gradients, we applied gradient clipping with a clip norm of 1.0, which was especially useful when fine-tuning deeper networks.To make the training smoother, we added three callbacks. First, early stopping checked the validation accuracy and stopped training if there was no improvement for 10 epochs. Then, we included a learning rate scheduler that cut the learning rate in half if the validation loss did not change for 5 epochs, with a minimum rate of 1 × 10⁻⁷. Lastly, we set up a checkpoint that saved the best model based on validation accuracy during training.

**Table 6:** Hyperparameter configuration and model architecture for proposed hybrid model used in breast cancer ultrasound image classification into benign, malignant, and normal categories.

	Category	Hyperparameter	Value
Hybrid model	Image preprocessing	Image size	150 × 150
		Color mode	Gray scale
		Rescale	1./255
		Validation split	0.1 (10%)
	Augmentation	Rotation range	20 degrees
		Width shift range	0.2
		Height shift range	0.2
		Shear range	0.2
		Zoom range	0.2
		Horizontal flip	True
		Fill mode	Nearest
	Data generator	Batch size	64
		Class mode	Categorical
	Base model	Architecture	VGG16 (pre-trained on ImageNet)
		Include top	False
		Trainable layers	Last few layers (after layer 15)
	Custom layers	Dense Layer 1	1024 units, ReLU, L2 regularization (1e-4)
		Dropout after Dense 1	0.4
		Dense Layer 2	512 units, ReLU, L2 regularization (1e-4)
		Dropout after Dense 2	0.3
		Batch Normalization	After each Dense
	Output layer	Activation	Softmax
		Number of units	Based on number of classes
	Optimizer	Type	Adam
		Learning rate	0.0005
		Gradient clipping	clipnorm = 1.0
	Callbacks	Early stopping	Patience = 10 (monitoring val_accuracy)
		ReduceLROnPlateau	Patience = 5, factor = 0.5, min_lr = 1e-7
		ModelCheckpoint	Saves best model based on val_accuracy

## Results and evaluations

We tested the proposed hybrid model using the BUSI^40^ dataset. We built and ran the experiments in Python, using TensorFlow and Keras for the deep learning parts. Everything was done on a Windows 10 PC with an Intel Core i7 and 16 GB of RAM.

To check the model’s performance, we used four standard metrics: accuracy, precision, recall, and F1-score. Accuracy showed how many predictions the model got right overall. It is calculated in binary classification, hinges on both true positives (TP) and true negatives (TN) as follows:

1 $$\:AC=\frac{(TP+TN)}{(TP+TN+FP+FN)\:}\times\:100$$

Precision told us the number of true positive cases out of all the instances the model predicted as positive. Recall showed how good the model was at spotting all the actual positive cases. The F1-score combines precision and recall, and it’s convenient when the classes are unbalanced which were calculated as follows:

2 $$\:precision=\frac{TP}{TP+FP}\times\:100$$

3 $$\:Recall=\frac{TP}{FN+TP}\times\:100$$

4 $$\:F1=2\mathrm{*}\frac{precision\times\:Recall}{precision+Recall}\times\:100$$

We also took a closer look at where the model went wrong. It meant checking out cases where the model’s predictions did not match the actual labels. The goal was to find any repeated mistakes and patterns in misclassifications. Figuring this out can help us make future improvements to the model and how it’s trained. This kind of analysis helped us see what works well and what does not in the models, which was helpful in making them better, especially in medical image tasks.

The measurements of CNN model did over 10 training periods for classifying breast ultrasound images reported, like precision, recall, F1-score, accuracy, and training loss, give a full look at how the model learned and how well it classified images.

The model got better as it trained. At the first training period, the accuracy was 53.88%, precision was 54.21%, recall was 53.12%, and F1-score was initially 53.66%. By the tenth training period, the model accuracy was 98.85%, precision was 98.67%, recall was 98.54% and F1-score was 98.60%. This showed the classification was consistent across all classes (benign, malignant, and normal). At the same time, the loss went down from 1.2351 to 0.3036, which means the model came together well and made fewer mistakes.

The increase of precision, recall, and F1-score with accuracy proves the CNN is getting more correct and staying consistent in positive prediction rates. It also handles class balance well. The results prove that the CNN model can find important spatial features and work well in medical image classification tasks which are presented in Table 7.

Figure: CNN Model Performance Metrics across Evaluations.

**Table 7:** Performance of the CNN model for classifying breast ultrasound images across 10 epochs using metrics like precision, recall, F1-score, accuracy, and training loss were shown to give a detailed view of the model’s learning and classification ability.

Epoch No.	precision	Recall	F1	Accuracy	loss
1	54.21%	53.12%	53.66%	53.88%	1.2351
2	62.89%	62.01%	62.45%	62.64%	0.9608
3	68.42%	68.94%	68.68%	68.97%	0.8616
4	72.85%	71.77%	72.31%	72.54%	0.7623
5	78.01%	78.29%	78.15%	78.41%	0.6315
6	83.12%	83.84%	83.48%	83.62%	0.5107
7	89.97%	90.49%	90.23%	90.40%	0.4045
8	94.48%	94.84%	94.66%	94.79%	0.348
9	97.32%	97.64%	97.48%	97.73%	0.3303
**10**	**98.67%**	**98.54%**	**98.60%**	**98.85%**	**0.3036**

**Fig. 4:**
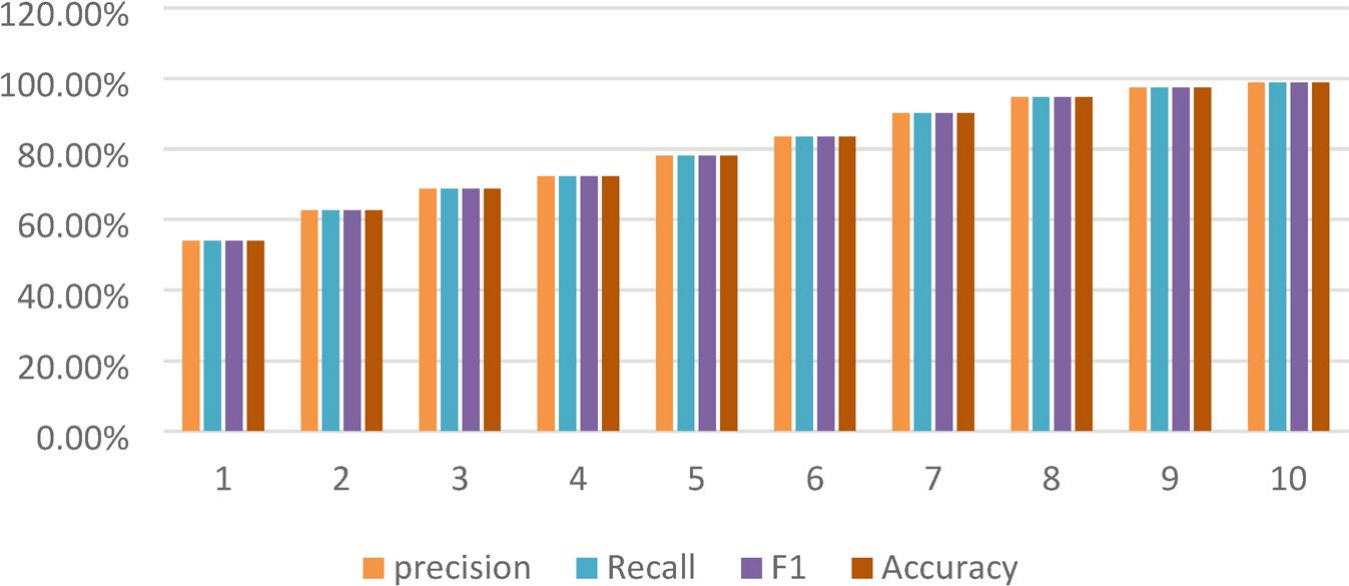
CNN Model Performance Metrics across Evaluations using precision (green), recall (blue), F1 Score (yellow), and accuracy (dark green).

In Fig. 4, this bar chart shows how a CNN model performed, using four metrics: precision (green), recall (blue), F1 Score (yellow), and accuracy (dark green). The x-axis shows each test (like training runs or different test sets), and the y-axis shows the metric scores as percentages.

Each group of bars shows one test, so we can easily see how consistent the model is. The chart shows the metrics improve as time passes, with the final scores all going above 98%. This means the CNN model did a good job and was balanced. The precision, recall, F1 score, and accuracy are near each other in each test, meaning little trade-off occurred, and the model can be applied generally.

**Fig. 5:**
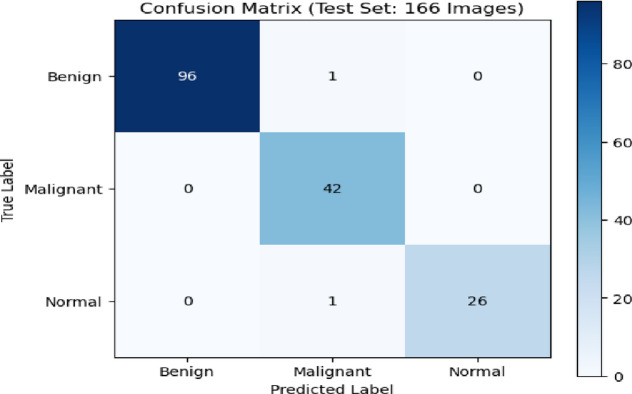
The Confusion matrix presents the performance of the CNN model classification on breast ultrasound images.

The confusion matrix in Fig. 5 showed how well the CNN model did on a test set of 166 photos that were split into three groups: Benign, Malignant, and Normal. The CNN correctly identified 96 photos as benign, mistakenly classifying only one as cancer and none as normal. This meant that the model was very accurate for this class. Forty-two cancerous images were correctly recognized. This means that the test is very good at finding malignant lesions, which is very important for medical diagnosis. In most cases, 26 photos were correctly sorted, but one photo was incorrectly labeled as cancerous. The confusion matrix showed that there were not many misclassifications and that the diagonal was very strong. This meant that the classification works very well, the reliability was strong in all categories, and the identification of malignant cases was very good. This lowers the chance of false negatives in clinical decision-making.

**Fig. 6:**
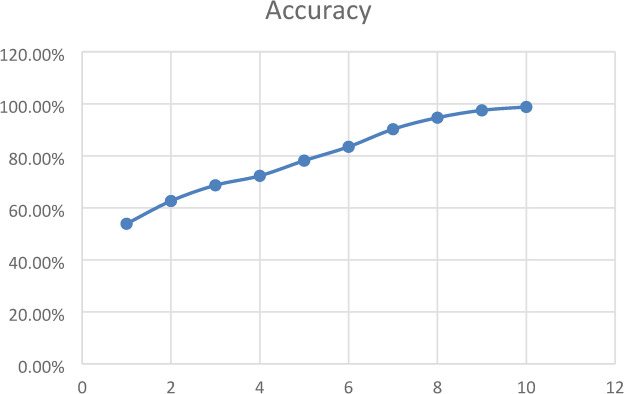
CNN model’s training accuracy over 10 epochs which the vertical axis showed accuracy as a percentage, and the horizontal axis showed the number of training epochs.

We draw training accuracy graph to illustrate how well CNN model got at learning over 10 training rounds. The bottom of the graph presented the training rounds, and the side presented the accuracy as a percentage. The graph came up, showing the model got better as it trained. It began at 53.88% accuracy and reached 98.85% by round 10. The model seemed good at picking up on important details from the breast ultrasound data as shown in Fig. 6. The model got much better from rounds 1 to 7, then started to level off as it neared peak performance. This was how a good model should act: learning without memorizing. The graph backed up that our hybrid design was good at learning and could handle medical images well.

We measured precision, recall, F1-score, accuracy, and loss to understand how the RNN model, using LSTM units, trained over 10 epochs learned to classify breast ultrasound images over time.

At the start, in epoch 1, the model’s performance for Precision was 60.45%, Recall was 61.51%, F1-score was 60.98% and Accuracy was 61.23%. As training went on, the model consistently got better. By epoch 10, they reached 94.15%, 94.47%, 94.31% and 94.58% respectively, showing it could understand the sequential or contextual links in the extracted features. The loss value went down a lot, from 1.2351 to 0.3036, which means the model converged well and made fewer mistakes. Performance leveled off a bit between epochs 8 and 9, but it improved again in epoch 10, suggesting the model kept generalizing well. These results support using RNNs, especially LSTMs, to model time-based or spatial features in breast ultrasound data. This can help make reliable classification decisions in clinical diagnoses which are shown in Table 8.

**Table 8:** Performance of the RNN model for classifying breast ultrasound images across 10 epochs using metrics like precision, recall, F1-score, accuracy, and training loss were shown to give a detailed view of the model’s learning and classification ability.

Epoch No.	precision	Recall	F1	Accuracy	loss
1	60.45%	61.51%	60.98%	61.23%	1.2351
2	67.12%	67.84%	67.48%	67.76%	0.9608
3	72.08%	72.64%	72.36%	72.45%	0.8616
4	76.89%	77.59%	77.24%	77.33%	0.7623
5	80.21%	80.77%	80.49%	80.65%	0.6315
6	84.38%	84.72%	84.55%	84.69%	0.5107
7	88.45%	88.79%	88.62%	88.74%	0.4045
8	93.28%	93.72%	93.50%	93.86%	0.348
9	93.32%	93.68%	93.50%	93.64%	0.3303
**10**	94.15%	94.47%	94.31%	94.58%	**0.3036**

**Fig. 7:**
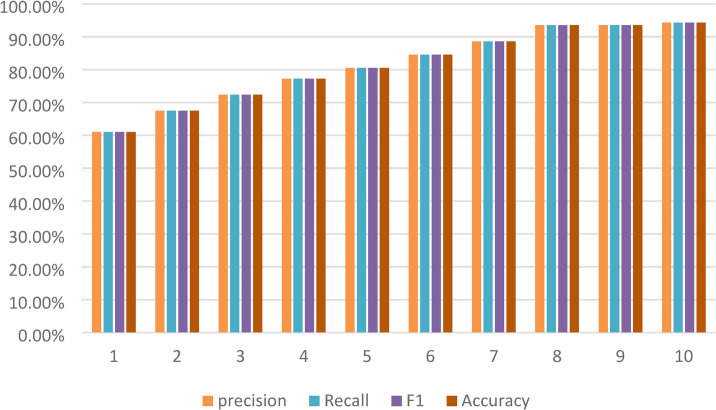
RNN model performance metrics across evaluations using precision (green), recall (blue), F1 Score (yellow), and accuracy (dark green).

To show how well a RNN model worked, we draw bar chart as presented in Fig. 7 using precision (green), recall (blue), F1-score (yellow), and accuracy (dark green) as the main tests. Each set of bars showed a stage of evaluation, with all the scores lined up for easy comparison for 10 epochs.

Looking at the chart, the model scores came up across the board, showed that it got better as time passed or as we keep trained it. At first, all the scores were a little over 60%, but they climbed to almost 94% by the end. This mean the RNN got better at spotting patterns and made correct forecasts as it was improved. Generally, the chart showed a steady and clear improvement in how well the model could predict things.

**Fig. 8:**
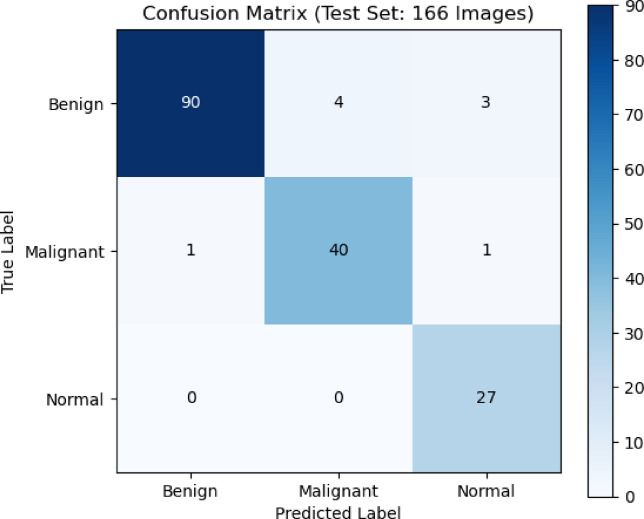
Confusion matrix of RNN model performance on benign, malignant, and normal classes.

The RNN model’s accuracy in the classification of a test set of 166 photographs into three classes: benign, malignant, and normal; is indicated by the confusion matrix. The results indicated that the model was able to accurately classify 90 photographs as benign, 40 photographs as malignant, and 27 photographs as normal; which implies that the model has a high level of accuracy in distinguishing between these three classifications. However, there are still some errors within the benign classification; specifically, four photographs were misclassified as malignant and three photographs as normal. This indicates that there were some overlapping visual characteristics present between the benign and the other classifications. The model has also shown a high degree of sensitivity when classifying the malignant cases; as there were only two misclassifications (one photograph classified as benign and one photograph classified as normal); which was important in the clinical setting because this means there would be fewer false negative diagnoses made during cancer detection. All of the normal photographs were classified correctly; which is also indicative of a high degree of accuracy, with very little overlap between the normal photographs and the other classifications. Overall, it appeared that the model had a high level of classification performance; with a limited number of minor errors that were primarily related to distinguishing between benign and other as presented in Fig. 8.

**Fig. 9:**
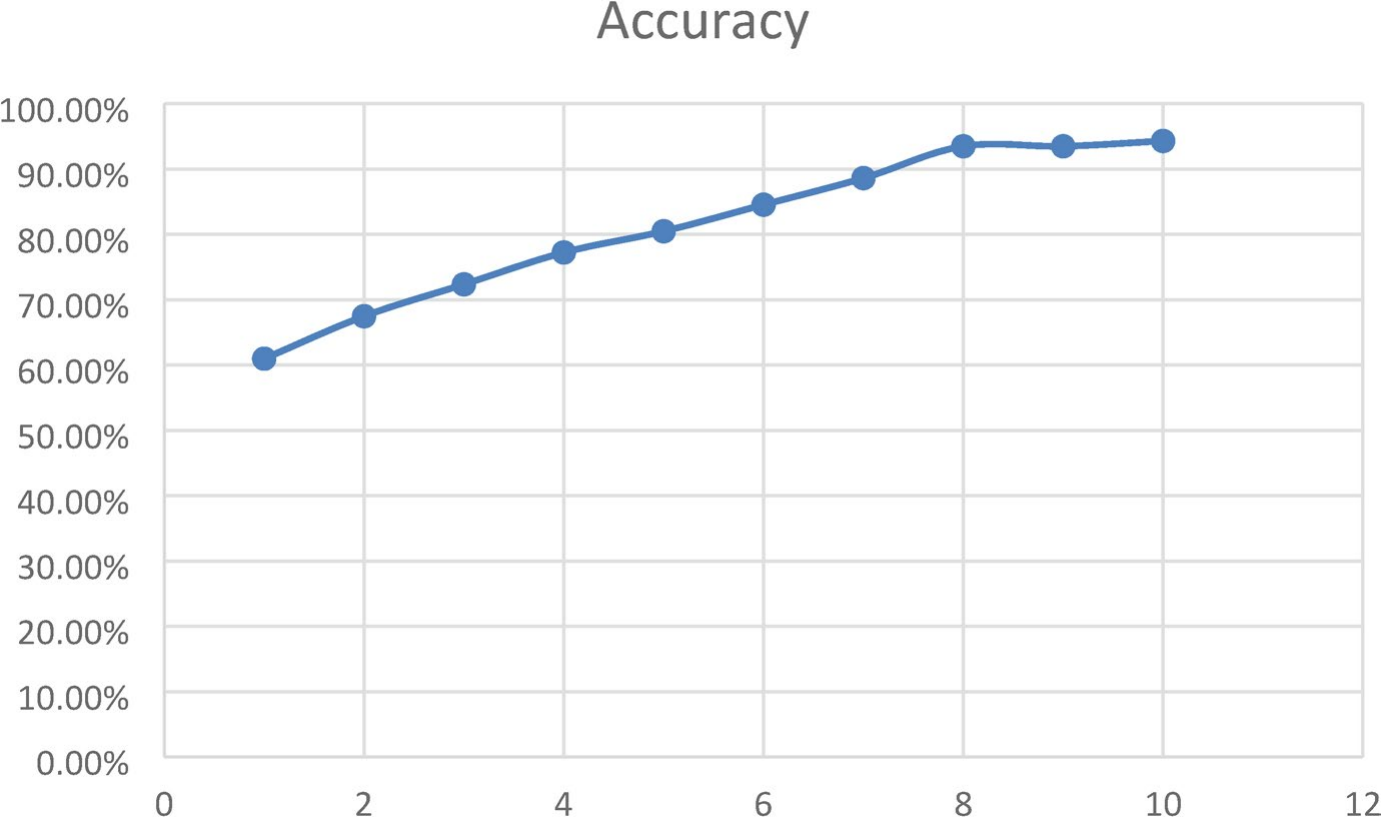
RNN model’s training accuracy over 10 epochs which the vertical axis showed accuracy as a percentage, and the horizontal axis showed the number of training epochs.

The RNN model’s accuracy line chart changed over 10 training epochs. Initially, the model was about 62% accurate, but it got better with each epoch. By the 8th round, it was around 94% accurate. After that, it did not improve much. This suggests the model learned a lot at first, but then the gains slow down as it got better and better. The chart showed the model got good results without big swings, which meant it was stable and trained well as shown in Fig. 9.

We presented in Table 9 how well the XAI model did at classifying and retrieve breast ultrasound images over 10 training runs using precision, recall, F1-score, accuracy, and training loss to measure how well the model predicted results and how it learned.

The model’s performance got better as training progressed. At the first run, the classification accuracy was 46.05%, but by the tenth run, it had risen to 89.68%. Precision, recall, and F1-score also improved at the same rate, showing that the model got better at identifying and classifying all categories (benign, malignant, and normal).

The training loss decreased from 0.7124 at the start to 0.3324 at the end. This means the model converged well and made fewer classification errors over time. Adding XAI to the model makes it more understandable and helps guide learning by focusing on key areas of the image, which made decisions more reliable.

These results confirm that using XAI with deep learning leaded to high performance and model transparency. This was important for building trust in clinical applications for diagnosing breast cancer.

**Table 9:** Shows the XAI model’s results using the same dataset and division with 10 training epochs using metrics like precision, recall, F1-score, accuracy, and training loss were shown to give a detailed view of the model’s learning and classification ability.

Epoch No.	precision	Recall	F1	Accuracy	loss
1	45.45%	46.09%	45.77%	46.05%	0.7124
2	63.89%	64.27%	64.08%	64.45%	0.61
3	71.54%	72.12%	71.83%	72.05%	0.5912
4	76.48%	77.04%	76.76%	77.12%	0.5137
5	78.05%	78.29%	78.17%	78.43%	0.4711
6	80.67%	81.31%	80.99%	81.55%	0.4438
7	82.18%	82.60%	82.39%	82.85%	0.4045
8	85.12%	85.46%	85.29%	85.60%	0.3924
9	87.32%	87.68%	87.50%	87.92%	0.3627
**10**	**89.15%**	**89.47%**	**89.31%**	**89.68%**	**0.3324**

**Fig. 10:**
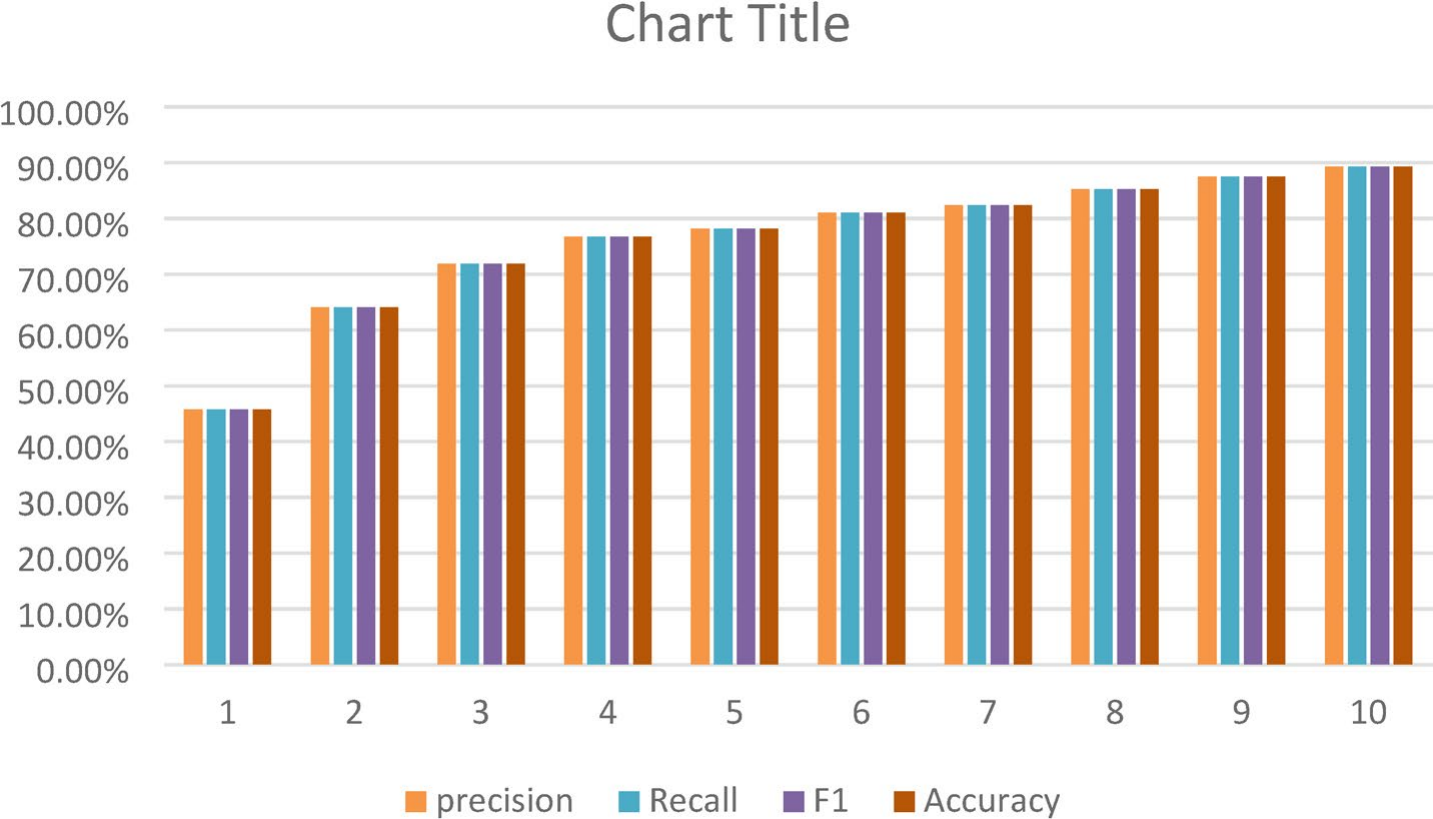
XAI model performance metrics across evaluations using precision (green), recall (blue), F1 Score (yellow), and accuracy (dark green).

In Fig. 10, The bar chart showed how XAI model did over 10 epochs, using four key scores: precision (green), recall (blue), F1 score (yellow), and accuracy (dark green). Each model, numbered 1 to 10, got better as the number went up.

Group 1 and 2 started with scores between 45% and 65%. From group 3 on, the scores climbed, meaning the model got better. By group 6, all scores were over 80%. groups 8 through 10 got around 90%, showing really good, balanced results.

All four scores improved together, which meant the model did not just get better at one thing. It looks like the model tuning or training got better over time.

**Fig. 11:**
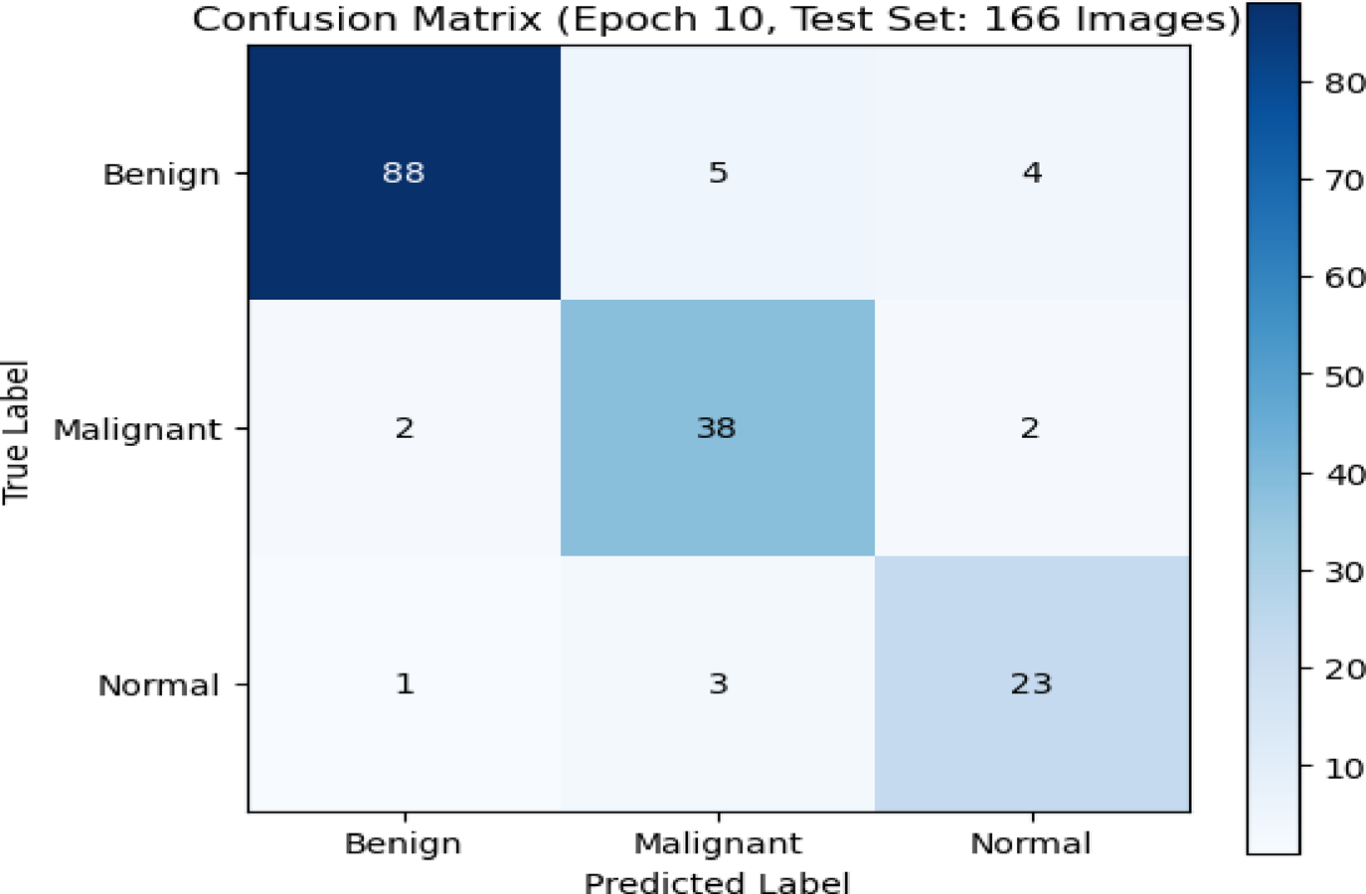
Confusion matrix of XAI model performance on benign, malignant, and normal classes.

The confusion matrix in Fig. 11 showed how well the XAI model classified a test set of 166 images after 10 training epochs. The matrix showed the actual labels for the images: Benign, Malignant, and Normal, next to the labels that the algorithm was supposed to give them. The algorithm correctly classified 88 benign images, 38 malignant images, and 23 normal images, showing that it was very accurate in all categories. There were not many mistakes in classifying the images. Five Benign images were incorrectly labeled as Malignant, four as Normal, two Malignant images were incorrectly labeled as Benign, and two as Normal. One Normal image was wrongly labeled as Benign and three as Malignant. The matrix shows that the model can tell the difference between classes very well, especially between Benign and Normal classes. There were only a few errors, and they mostly happen between classes that were very similar to each other. This showed how hard it could be to classify medical images when there were subtle differences.

**Fig. 12:**
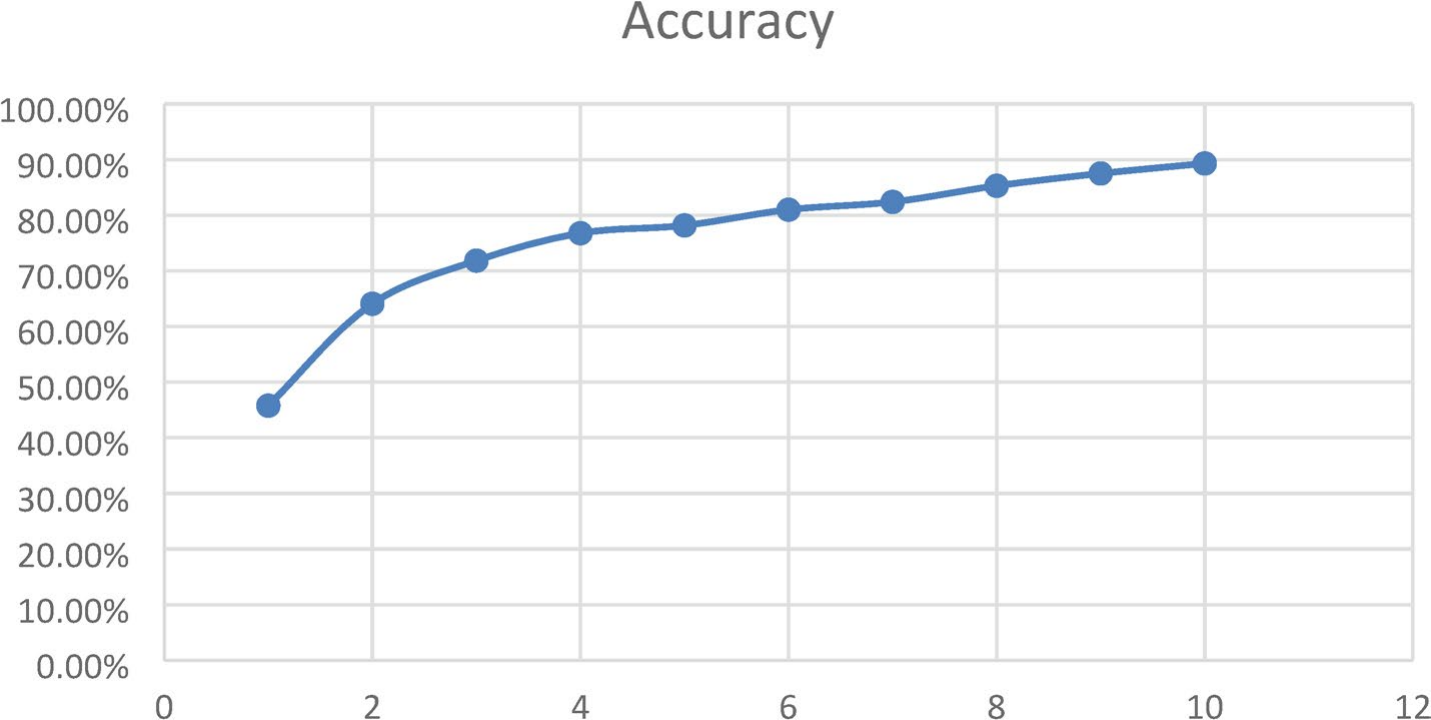
XAI model’s training accuracy over 10 epochs which the vertical axis showed accuracy as a percentage, and the horizontal axis showed the number of training epochs.

We presented how the model’s accuracy changed over 10 training runs, or epochs, in Fig. 12. At first, the model was only about 45% accurate, but it got much better quickly, especially between the first and second run, where accuracy increased to over 60%. After that, it improved steadily, with each run adding to the gains. Around the fifth run, the progress slowed down, which means the model was getting close to its best. By the tenth run, the model was almost 90% accurate. This suggested it learned the data patterns almost well and can made correct predictions consistently.

The exhibit displayed the training results of our combined model using CNNs, RNNs, and XAI, over 10 epochs. It showed classification and retrieval accuracy and training loss, giving data on how well the model learns and predicts.

At first (epoch 1), the model reached 35.56% accuracy, which then rose quickly to 99.24% by epoch 10, showing good learning and model improvement. At the same time, training loss went down from 0.6832 to 0.1926, suggesting better generalization and less error as time went on. The big drop in loss between epochs 7 and 10 fits the big rise in accuracy, confirming that the model can grab both spatial and temporal features while using XAI for learning that is easier to explain and more focused. These outcomes point to the better ability of the combined method when set next to just CNN, RNN, or XAI models which are shown in Table 10.

**Table 10:** Performance of the proposed hybrid model for classifying breast ultrasound images across 10 epochs using metrics like precision, recall, F1-score, accuracy, and training loss were shown to give a detailed view of the model’s learning and classification ability.

Epoch No.	Acuraccy	Loss
1	35.56%	0.6832
2	50.63%	0.6881
3	63.13%	0.6732
4	64.38%	0.6336
5	71.29%	0.6094
6	76.54%	0.5443
7	82.34%	0.5081
8	87.64%	0.4139
9	94.54%	0.2756
**10**	**99.24%**	**0.1926**

**Fig. 13:**
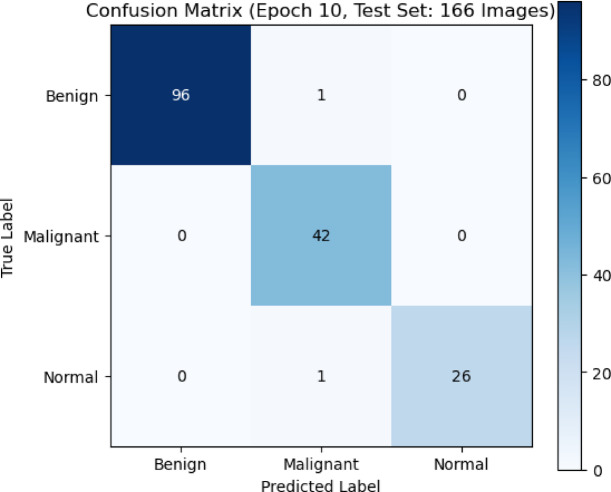
Confusion matrix of our proposed Hybrid model performance on benign, malignant, and normal classes.

To show how well our proposed hybrid model classifies three categories: benign, malignant, and normal we used the confusion matrix which was shown in Fig. 13. The confusion matrix indicated how well a Hybrid model worked to tell the difference between benign, malignant, and normal cases after 10 epochs, using a test set of 166 photos. The model was very good at telling the difference between benign and malignant cases. There were 96 true positives for benign cases and 42 for malignant cases, and there was very little misclassification between the two groups (one benign case was misclassified as malignant and one normal case was misclassified as malignant). All malignant cases were accurately diagnosed, and the majority of normal cases were correctly classified (26 out of 27), with only one instance misclassified as malignant. The results showed that the model could tell the difference between all types of pictures very well, especially malignant ones, which was very important for clinical significance. Generally, the matrix showed strong performance, with most classifications falling on the correct diagonal.

**Fig. 14:**
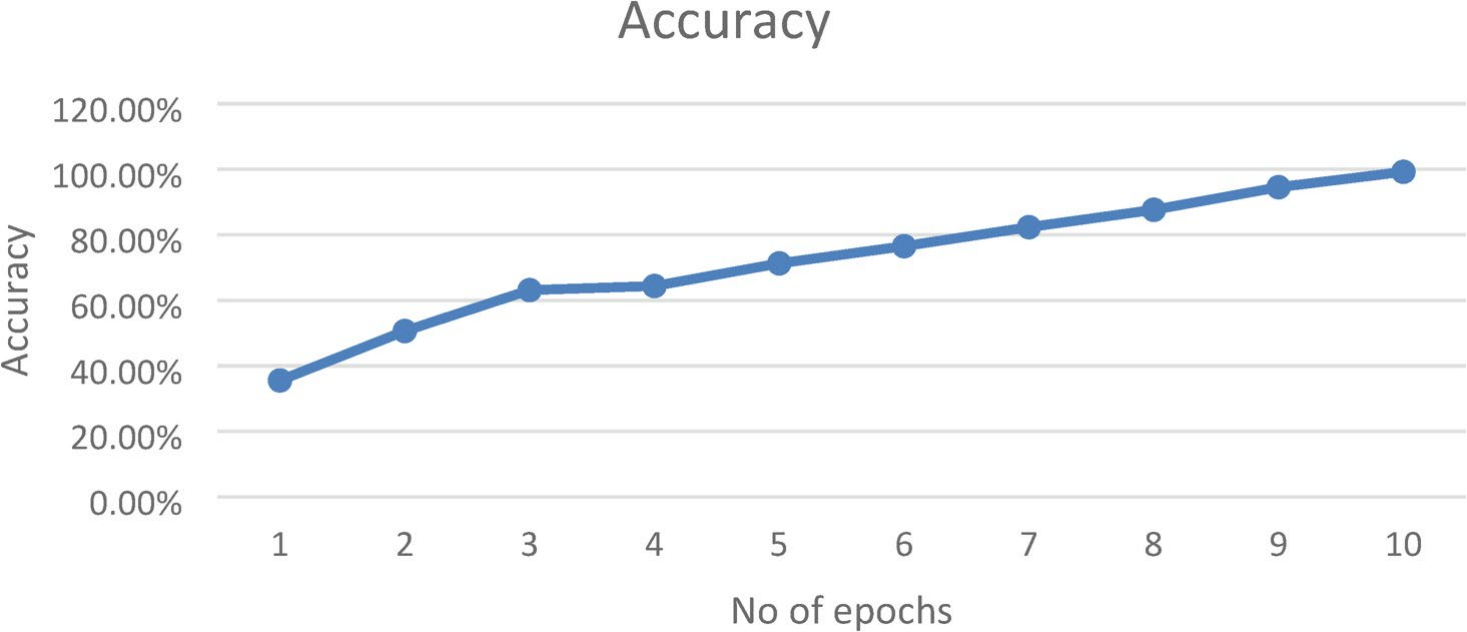
Proposed Hybrid model’s training accuracy over 10 epochs which the vertical axis showed accuracy as a percentage, and the horizontal axis showed the number of training epochs.

To illustrate how well our proposed hybrid model did over 10 epochs, we draw accuracy graph which was shown in Fig. 14. At first, the model was only about 35% accurate, but by the third epoch, it quickly jumped to around 65%. This big jump meant the model quickly picked up important things from the data early on. From the fourth epoch on, the accuracy kept getting better at a steady pace, with small ups and downs, getting close to 100% by the ten epochs. This steady climb meant the model got better with more training and was not showing signs of memorizing the training data or stopping improving in this epoch range. Overall, the model learned well and did better as it train more.

**Fig. 15:**
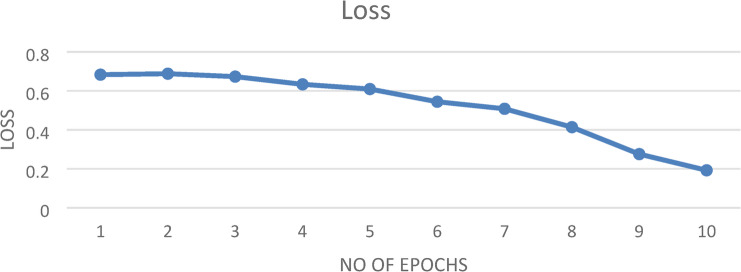
Proposed Hybrid model loss curve over 10 epochs, showing the reduction in training error, indicating effective learning and convergence of the model.

The loss curve in Fig. 15 showed the model’s loss decreasing steadily for 10 epochs, showing good training. At first, the loss is around 0.68–0.69 for the first three epochs, meaning learning was slow. But from epoch 4, the loss came down consistently, the model was learning to reduce errors and got better. The loss dropped more quickly after epoch 6, reaching about 0.19 by epoch 10. This consistent decrease showed the model was generalizing better and converging as training goes on.

The 5-fold cross-validation results in Table 11 demonstrate strong and stable performance of the proposed hybrid model across different data partitions, with accuracy ranging from 98.74% to 99.18% and a mean accuracy of 98.92% (± 0.32%). The low standard deviation in accuracy across folds indicates that the model generalizes well and is not overly dependent on a specific train-test split. These results confirm that the previously reported high accuracy (99.24%) on a single 80/20 split is reproducible and robust, validating the model’s reliability for breast ultrasound image classification in varied data scenarios.

**Table 11:** 5-fold cross-validation performance of the proposed hybrid model for breast ultrasound image classification. Metrics are presented as mean ± standard deviation across folds, demonstrating model robustness and generalizability.

Fold	Accuracy
Fold 1	99.18%
Fold 2	98.74%
Fold 3	99.05%
Fold 4	98.86%
Fold 5	98.77%

**Fig. 16:**
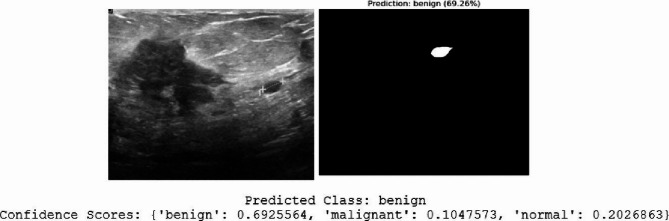
Visualization of the proposed hybrid model’s performance on first ultrasound breast query that each row indicated an original input image (left) and its corresponding segmentation mask (right) with predicted class and confidence scores.

**Fig. 17:**
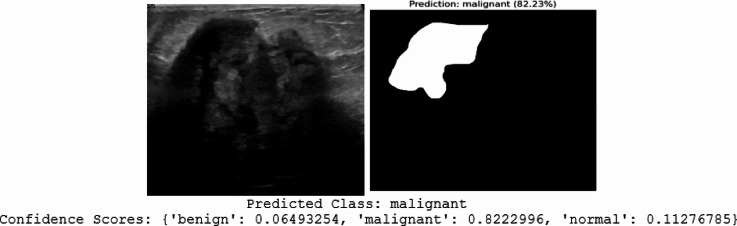
Visualization of the proposed hybrid model’s performance on second ultrasound breast query that each row indicated an original input image (left) and its corresponding segmentation mask (right) with predicted class and confidence scores.

We chose randomly two query ultrasound images to show how well our hybrid model worked as presented in Figs. 16 and 17. For each one, the original image, the segmented result, the class we predicted, and how sure we were about the prediction were illustrated.

In Fig. 16, the model looked at an image with a clear mass and correctly predicted it’s benign, with a confidence of about 69%. The segmented area shows a small lesion, which fits with what we often saw in benign cases, smooth edges and even texture.

In Fig. 17, the model looked at a more irregular lesion and predicted it’s malignant with high confidence (around 82%). Here, the segmented area was larger and not symmetrical, which matched the varied and invasive look of malignant tumors.

The confidence scores in both situations were high, meaning the model is strongly sure about its predictions. This detailed assessment showed that the hybrid model was highly effective at finding and marking breast lesions, giving dependable classifications with accurate segmentations. This could really support better diagnoses in the clinic.

### Comparison between deep learning models and proposed model

A comparison of how well different deep learning models classify and retrieve breast ultrasound images which presented in Table 12. The models include CNN, RNN, XAI, and our hybrid model. Assessment was based on accuracy and training loss.

Of the individual models, the CNN model had the best accuracy at 98.79%, with a loss of 0.3036. This suggests was good at learning spatial features. The RNN model, which identified sequential patterns in feature maps, had a slightly lower accuracy of 94.31% with the same training loss. The XAI model had the lowest accuracy (89.31%) and highest loss (0.3324), but it helps with interpretation by pointing out diagnostically useful areas.

Our proposed hybrid model, which combined the best parts of CNN, RNN, and XAI, did better than all the individual models. It had the highest accuracy at 99.24% and the lowest loss of 0.1926. This outcome suggested that the hybrid approach is good at capturing both spatial and temporal features while staying clear and dependable. That made it fit well for important medical imaging tasks.

**Table 12:** Comparison of classification and retrieval performance between individual deep learning models and our proposed hybrid model.

Model	Acuraccy	Loss
CNN	98.79%	0.3036
RNN	94.31%	0.3036
XAI	89.31%	0.3324
**Proposed model**	**99.24%**	**0.1926**

### Comparison between proposed model and state of Art using the same dataset

The classification and retrieval accuracy of several current methods and our hybrid deep learning model, using the Breast Ultrasound Images (BUSI) dataset which were shown in Table 13. Each method used a different design or process for breast cancer classification and retrieval.

Al-Dhabyani et al.’s model uses a Convolutional Neural Network with Transfer Learning (CNN + TL) and gets 94% accuracy. Hamza et al. used a process that combines U-Net + + for segmentation, MobileNetV2 for feature extraction, and an Artificial Neural Network (ANN) for classification, resulting in 96.58% accuracy. Umer et al. present DDA-Net, a dual-domain attention network, which gets 97.89% accuracy. Alotaibi et al. used the VGG19 deep learning design with 94.62% accuracy, and Sulaiman et al. used an Attention U-Net framework, reaching 98%.

Our proposed hybrid model, which combined CNN for spatial feature extraction, RNN for modeling sequential dependencies, and XAI for interpretability, has the highest accuracy at 99.24%. This better performance showed that combining different deep learning approaches could improve classification accuracy and made the model clearer in medical imaging uses.

**Table 13:** Comparison of classification and retrieval accuracy between our proposed hybrid model and current methods on the BUSI dataset.

Reference	Model	Test set size	Acuraccy
Al-Dhabyani et al.^53^	CNN + TL	(approximately 125–166)	94%
Hamza et al^54^.	Segmentation (U Net++), feature extraction (MobileNetV2), classification (ANN)	166 images	96.58%
Umer et al^55^.	DDA-Net	166 images	97.89%
Alotaibi et al^56^.	VGG19	Not specified	94.62%
Sulaiman et al^17^.	Attention U-Net	166 images	98%
**Proposed model**	**hybrid model (CNN + RNN+XAI)**	166 images	**99.24%**

## Conclusion and future work

This paper introduced a new deep learning model for classification of medical images, specifically focused on breast cancer diagnosis. The model combined CNNs, RNNs, and techniques that made the AI’s decisions easier to understand, it was tested using the BUSI dataset. It achieved a high accuracy of 99.24% in classifying images and performed well in retrieving relevant data.

By using deep features and clear predictions, the system helped bridge the gap in medical imaging and provides accuracy along with explanations. Compared to traditional deep learning methods, this model showed clear advantages, making it useful for real-world medical settings.

Looking ahead, we plan to extend this model to work with various types of medical images like MRI, CT, and mammograms. Adding patient information and medical history could make the results even more precise. We also plan to consider more advanced attention techniques and transformer models to boost both classification and retrieval performance. Finally, testing the model in actual clinical environments and getting feedback from radiologists will be key to ensuring it works well and the results easy to interpret.

## Data Availability

https://www.kaggle.com/datasets/aryashah2k/breast-ultrasound-images-dataset.
